# Breathing as a Fundamental Rhythm of Brain Function

**DOI:** 10.3389/fncir.2016.00115

**Published:** 2017-01-12

**Authors:** Detlef H. Heck, Samuel S. McAfee, Yu Liu, Abbas Babajani-Feremi, Roozbeh Rezaie, Walter J. Freeman, James W. Wheless, Andrew C. Papanicolaou, Miklós Ruszinkó, Yury Sokolov, Robert Kozma

**Affiliations:** ^1^Department of Anatomy and Neurobiology, University of Tennessee Health Science CenterMemphis, TN, USA; ^2^Department of Pediatrics, Division of Pediatric Neurology, University of Tennessee Health Science Center and Le Bonheur Children’s Hospital Neuroscience InstituteMemphis, TN, USA; ^3^Department of Molecular and Cell Biology, Division of Neurobiology, University of California at BerkeleyBerkeley, CA, USA; ^4^Rényi Institute of Mathematics, Hungarian Academy of SciencesBudapest, Hungary; ^5^Department of Mathematical Sciences, University of MemphisMemphis, TN, USA; ^6^Department Computer Sciences, University of Massachusetts AmherstAmherst, MA, USA

**Keywords:** mind-body, cortical oscillations, respiration, embodied cognition, phase transitions, phase amplitude coupling, proprioception, graph theory

## Abstract

Ongoing fluctuations of neuronal activity have long been considered intrinsic noise that introduces unavoidable and unwanted variability into neuronal processing, which the brain eliminates by averaging across population activity (Georgopoulos et al., 1986; Lee et al., 1988; Shadlen and Newsome, 1994; Maynard et al., 1999). It is now understood, that the seemingly random fluctuations of cortical activity form highly structured patterns, including oscillations at various frequencies, that modulate evoked neuronal responses (Arieli et al., 1996; Poulet and Petersen, 2008; He, 2013) and affect sensory perception (Linkenkaer-Hansen et al., 2004; Boly et al., 2007; Sadaghiani et al., 2009; Vinnik et al., 2012; Palva et al., 2013). Ongoing cortical activity is driven by proprioceptive and interoceptive inputs. In addition, it is partially intrinsically generated in which case it may be related to mental processes (Fox and Raichle, 2007; Deco et al., 2011). Here we argue that respiration, via multiple sensory pathways, contributes a rhythmic component to the ongoing cortical activity. We suggest that this rhythmic activity modulates the temporal organization of cortical neurodynamics, thereby linking higher cortical functions to the process of breathing.

We have recently shown that respiration-locked olfactory bulb activity in awake, head restrained mice causes respiration-locked delta oscillations and gamma power modulations in the somatosensory cortex (Ito et al., 2014). This unexpected direct ability of respiration-locked sensory activity to modulate oscillatory neuronal activity in the neocortex led us to consider the potential wider implications of a link between breathing and brain activity, particularly with respect to the possibility that respiration influences cortical neuronal activity underlying cognitive function.

Based on our own experimental findings, results from our modeling studies using a simple graph theory model and a review of the literature, we argue that respiration, via multiple sensory pathways, provides a subtle but continuous rhythmic modulation of cortical neuronal activity that modulates sensory, motor, emotional and cognitive processes. Specifically, we hypothesize that: (1) respiration causes respiration-locked oscillations that are synchronized across large areas of neocortex at the species-specific respiratory rhythm; (2) that increases in the power of gamma oscillations (40–100 Hz) occur preferably during certain phases of (i.e., are phase-locked to) the respiratory cycle. Both hypotheses are supported by solid experimental results in the somatosensory barrel cortex in awake mice (Ito et al., 2014; Figure 1) and by our modeling studies (see below). Additional results, published in abstract form, support the possibility that respiration-locked oscillations are also present in several other areas of mouse neocortex (Liu et al., 2015), including the visual cortex (McAfee et al., 2016).

**Figure 1:**
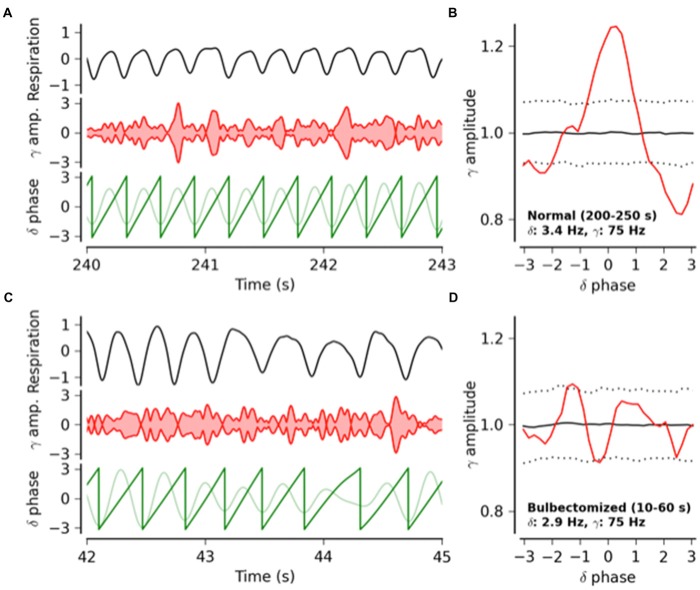
**From Ito et al. (2014): respiratory modulation of the power of gamma frequency oscillations in mouse whisker barrel cortex.** Phase–amplitude coupling between respiration-locked delta and gamma band oscillations in the barrel cortical local field potential (LFP) activity of an awake intact and an awake bulbectomized mouse, followed by population statistics. **(A)** Respiratory activity (top trace), amplitude of gamma band oscillations (middle trace) and delta oscillations (light green bottom trace) and its phase (dark green bottom trace) in an intact mouse. Gamma oscillation (75 Hz) amplitude peaks rhythmically phase locked to the delta cycle. **(B)** Gamma oscillation amplitude as a function of delta phase (red). The solid and dotted black lines indicate the mean and the 2.5 and 97.5 percentile boundaries of the surrogate amplitude distribution estimated from 1000 phase-randomized surrogates. Gamma amplitude modulation is significant at phase 0 of the delta cycle. **(C,D)** Same as **(A,B)**, respectively, but for a bulbectomized mouse. After removal of the olfactory bulb, the amplitude modulation of the gamma band oscillations is no longer phase locked to respiration.

A third prediction is supported by preliminary experimental findings on sudden changes in synchronization patterns of neural activity published in abstract form (Kozma et al., 2015). We predict that (3) the timing of the sudden changes in the network activity, i.e., fast transitions between synchronized and de-synchronized network states, are phase-locked to the respiratory rhythm. These transitions can be detected as jumps in the analytic phase of oscillatory population activity using a Hilbert transform based analysis of local field potential (LFP) or electroencephalographic (EEG) activity (Freeman and Rogers, 2002; Freeman et al., 2006; Freeman, 2015). Graph theoretical arguments provide a modeling framework to describe the experimentally observed sudden changes as “phase transitions” (Puljic and Kozma, 2008; Kozma and Puljic, 2015), a term we will use throughout this article.

As we lay out in more detail below, gamma oscillations are forms of cortical activity widely linked to cognitive and other higher cortical functions. Our hypotheses predict that a consciously controlled change in respiratory behavior will cause a change in cognitive and emotional states, which is a common observation in yogic breathing (Jella and Shannahoff-Khalsa, 1993; Stancák and Kuna, 1994; Brown and Gerbarg, 2005) and stress reducing respiratory exercises such as combat tactical breathing employed by military and special forces (Grossman and Christensen, 2011). A second key prediction is that respiration-locked modulation of cortical gamma activity and phase transition timing directly links respiratory behavior to higher cortical processes, including cognitive and limbic functions, sensory perception and motor control. The respiration-locked modulation of neocortical activity we propose here would thus provide a neuronal mechanism and causal link between respiration and pain perception (Arsenault et al., 2013; Iwabe et al., 2014), motor control (Ebert et al., 2002; Rassler and Raabe, 2003; Li and Laskin, 2006; Iwamoto et al., 2010; Cao et al., 2012; Krupnik et al., 2015), attention (Gallego et al., 1991; Krupnik et al., 2015) and emotion (Benson et al., 1974; Arch and Craske, 2006; Homma and Masaoka, 2008).

Oscillations of neocortical activity in the gamma (30–100 Hz) frequency range, have been strongly implicated in affective and cognitive brain functions such as attention (Fries et al., 2001; Laufs et al., 2003; Tallon-Baudry, 2004), sensory perception (Engel et al., 2001; Tallon-Baudry, 2003; Gould et al., 2012), decision making (Kay and Beshel, 2010; Siegel et al., 2011; Gould et al., 2012; van Vugt et al., 2012; Wyart et al., 2012; Nácher et al., 2013), problem solving (Sheth et al., 2009), memory formation (Marshall et al., 2006; Tort et al., 2009; Chauvette et al., 2012) and language processing (Crone et al., 2001; Towle et al., 2008; Babajani-Feremi et al., 2014).

Sudden changes in network synchronization are characteristic features of cortical activity that have been widely linked to cognitive processes (Kozma and Freeman, 2016). Detailed analysis of rabbit and human intracranial electrocorticography (ECoG) signals revealed discontinuities in the analytic phase determined by Hilbert analysis (Freeman and Rogers, 2002; Freeman et al., 2006; Freeman, 2015). Experiments with rabbits trained with a classical conditioning paradigm showed that discontinuities of the analytic phase have cognitive relevance (Freeman, 2004; Kozma and Freeman, 2008). Namely, after delivering the conditioned stimulus, the occurrence of the phase discontinuity correlates with the stimulus, suggesting that these discontinuities can be viewed as markers of the cognitive activity (stimulus classification) performed by the rabbits.

Schölvinck et al. (2015) observed that variability of neuronal responses in the primary visual cortex to repeated identical stimuli was caused by large scale network activity, which was more variable when the network was in a synchronized state vs. an asynchronous state. Recently, Tan et al. (2014) also showed that visual stimulation shifted the activity states of the macaque primary visual cortex from synchronous to asynchronous activity. These findings are fundamentally in line with our hypothesis that the timing of such phase transitions is linked to the rhythmic sensory stimulation caused by respiration. We have obtained preliminary supporting evidence for phase-locking between respiration and phase discontinuities in human cortical activity from an analysis of ECoG signals from a human subject. We interpreted the results as phase transitions in cortical population activity between synchronized and de-synchronized states; see Kozma et al. (2015).

A small group of researchers have envisioned the possibility of respiration influencing large-scale brain activity via the olfactory system. Freeman and colleagues performed pioneering studies on the influence of respiration through olfaction on the rat brain (Eeckman and Freeman, 1990; Kay and Freeman, 1998). Effects of theta-modulation of saccadic signals have been described as visual sniffing (Kozma and Freeman, 2001). Fontanini and Bower (2006) speculated that olfactory bulb respiration-locked oscillations in rodents may propagate through the entire cortex. However, none of these earlier studies anticipated that respiration could modulate the power of gamma oscillations or considered a respiratory influence on the timing of phase transitions in cortical population activity as a mechanism that directly links respiratory behavior and cognitive brain processes.

Respiration creates both conscious and unconscious streams of rhythmic sensory inputs to the brain. Consciously accessible sensations of normal, unobstructed breathing include odor perception, the mechanical and thermal sensation of air flowing through nose, mouth and upper airways, and the proprioception of movements of the chest and abdomen. Unconscious sensory signals caused by respiration include interoceptive signals from the lungs, diaphragm and internal organs, which represent the mechanical consequences of respiratory movements, and the chemosensitive signals from the cardiovascular system, which represent breath-by-breath fluctuations of CO_2_ and O_2_ levels in the blood. The sensations and brain activity patterns associated with hunger-for-air (Liotti et al., 2001; Macey et al., 2005) are not considered here, as they represent an emergency response not related to normal, unobstructed breathing.

There are also a number of indirect ways cortical areas receive respiration-locked sensory input. Eye movements, for example, have been shown to be transiently phase-locked to respiration during sleep (Rittweger and Pöpel, 1998) as well as in the awake state (Rassler and Raabe, 2003). Recently, Ito et al. (2013) reported saccade related changes in the power of neuronal oscillatory activity in four frequency bands, including gamma, in primates that were freely viewing their environment. This suggests that the retinal flow associated with eye movements causes a modulation of power in visual cortical oscillations that is partially correlated with respiration. Another indirect respiration-locked sensory input comes from the auditory cortex, which receives rhythmic auditory input related to respiration caused by the sound of air flowing through the nose or mouth. Finally, neurons in the brain stem project broadly to thalamic nuclei (Carstens et al., 1990; Krout et al., 2002). These projections likely provide respiration-locked input to the thalamus (Chen et al., 1992), introducing a non-sensory respiratory rhythm to the thalamo-cortical network.

While there are many sources of respiration-locked activity, the olfactory system deserves special attention, because early mammals relied strongly on their olfactory sense and had proportionately large olfactory bulbs (Rowe et al., 2011). Furthermore, neuronal oscillations, particularly gamma oscillations, are a universal element of odor processing in animals as far removed from joint evolutionary ancestors as mammals and insects are (Kay, 2015). Even though in primates the olfactory sense lost the prime importance it has for most other mammals in favor of vision (Gilad et al., 2004). EEG studies comparing nasal and oral breathing of room air found that nasal breathing elicited significantly different patterns of EEG activity than mouth breathing (Servít et al., 1977; Lorig et al., 1988). This is in line with our findings of nasal air flow in mice driving delta oscillations and gamma power modulations in a non-olfactory area of neocortex (Ito et al., 2014) and suggests that the olfactory bulb activation exerts similar influence on human cortical activity.

The detection and analysis of respiration locked cortical activity requires the simultaneous measurement of respiration and brain activity. Such simultaneous measurements are not commonly performed. A notable exception is a recent study of the effects of sleep disordered breathing (SDB) in children on cortical oscillatory activity (Immanuel et al., 2014). Immanuel et al. (2014) showed that the average power of the EEG signal decreased during inspiration and increased during expiration, in a frequency band and sleep stage dependent manner, in both healthy subjects and subjects suffering from SDB. This study did, however, not evaluate phase-locking between EEG oscillations and the respiratory cycle.

Respiration related sensory activity during unobstructed breathing mainly reaches three areas of the cortex: (1) the olfactory cortex and surrounding areas receive olfactory bulb input; (2) the somatosensory cortex receives inputs from mechanoreceptors of chest, the abdominal skin and muscles that are stretched and moved by respiration; and (3) the insular cortex receives input from chemoreceptors and mechanoreceptors in the lungs, diaphragm and internal organs. Our recordings of olfactory bulb dependent respiration-locked oscillations in the mouse somatosensory cortex suggest that respiration-locked activity propagates from primary sensory areas to parts of the cortex that do not receive direct respiration related sensory inputs. A likely mode of propagation is through the cortico-cortical network itself, possibly involving also cortico-thalamic connections. However, the anatomy of axonal connections within the parabulbar and limbic areas suggest a number of subcortical regions and neuromodulator systems may also be influenced by respiration-driven sensory input. For example, widely projecting serotonergic and cholinergic neurons within the rat basal forebrain have been shown to rhythmically discharge in phase with respiration (Manns et al., 2003; Mason et al., 2007), with olfactory bulb respiration-locked activity as a likely driving force (Linster and Hasselmo, 2000). Stimulation of cholinergic neurons in particular is associated with increased neocortical gamma oscillations (Cape and Jones, 2000) a mechanism that might contribute to the respiration-locked modulation of gamma power in mouse somatosensory whisker barrel cortex (Ito et al., 2014). However, as we argue below, respiration-locked gamma power modulation may result from intrinsic properties of the cortical network itself.

The link between respiration-locked cortical oscillations and respiration-related sensory inputs to the cortex is straightforward: respiration-locked rhythmic inputs drive cortical neurons to fire rhythmically at the same frequency. Experiments in anesthetized rodents show that respiration-locked oscillations in the piriform cortex are driven this way by respiration-locked activity of olfactory bulb afferents (Fontanini and Bower, 2005; Uchida et al., 2014), which also drive respiration-locked activity in the hippocampus of mice, both under anesthesia (Yanovsky et al., 2014) and while awake and walking on a tread mill (Nguyen Chi et al., 2016). However, the mechanisms behind respiration-locked modulations of gamma power, which we observed in the mouse somatosensory cortex (Figure 1), are less obvious.

To investigate the processes leading to respiration locked increases in the power of gamma oscillations we used a simple graph theory model inspired by cortical network architecture, with a biologically appropriate balance of excitatory and inhibitory neurons and mix of short- and long-range connections. Expanding on previous work (Reijneveld et al., 2007; Turova and Villa, 2007; Gallos et al., 2012; Janson et al., 2016), we define a geometric graph, which is the combination of a regular 2-dimensional square lattice with N × N vertices, and a few additional long edges between some lattice points. The additional long edges, or “shortcuts” are selected randomly according to probability *p* = c/(N × d), where *d* is the Euclidian distance between the lattice points, and c is a constant (Janson et al., 2015). Note that this model defines a scale-free distribution of the shortcuts with power exponent 1. The expected number of long edges per node has been shown to be LAMBDA = 2c * ln(2). Next, we define an activation process on the random lattice graph, and the activation of a node at time *t* + 1 is denoted as *A*_v_(*t* + 1). Note that some of the nodes are excitatory (E), while others are inhibitory (I). In the present model, we select 25% of the nodes as inhibitory and the rest are excitatory. The update rule is defined by the so-called “k-majority”, i.e., a node becomes active at time *t* + 1, if more than *k* of its neighbors have been active at time *t*, while it will be inactive in the opposite case. Note that inhibitory nodes have inverse effects on excitatory nodes; namely, the activity of inhibitory nodes are subtracted from the total activation when the k-majority rule is tested (for details see Janson et al., 2015).

Our model has several parameters; the number of shortcuts (LAMBDA); the ratio of excitatory nodes (OMEGA), and threshold parameter (*k*). In a regular square lattice without shortcuts, the majority rule is given by *k* = 2. In the results shown here, we select *k* = 2 and *k* = 3 for E and I nodes, respectively. Figure 2A shows that depending on the choice of LAMBDA and OMEGA, various dynamical regimes can be modeled, such as limit cycle, non-zero fixed point (following a dampened oscillation), and zero fixed point.

**Figure 2:**
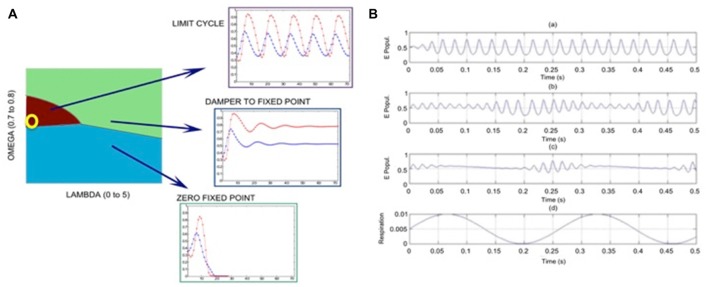
**Results of calculations using graph theory models of coupled excitatory-inhibitory populations; the following parameter values are used: proportion of excitatory units OMEGA = 0.75, expected number of long axonal connections (shortcuts) is LAMBDA = 0.0017. (A)** Phase diagram with parameter regions with the dominance of limit cycle oscillations (purple), nonzero fixed point (light green) and zero-fixed point (blue) regimes; the yellow circle corresponds to parameter settings used in **(B)** plot at the edge of the limit cycle regime, close the fixed point regime. **(B)** Illustration of the phase-locked amplitude modulation of the gamma oscillations (of excitatory population) in response to periodic input (respiration) perturbations of increasing amplitude (RA); **(Ba)** RA = 0.001; **(Bb)** RA = 0.02; **(Bc)** RA = 0.03; **(Bd)** shape of the respiratory sinusoid signal. The amplitude modulation of the inherent high-frequency oscillation (around 60 Hz) is locked to the respiratory cycle, so that the high-frequency component has increased magnitude during the increasing segment of the input signal from its minimum value.

In order to simulate respiratory effects, we introduce a sinusoidal input with magnitude (RA). In this model we select parameters OMEGA = 0.75 and LAMBDA = 0.0017; this parameter choice is illustrated by yellow circle in Figure 2A. Examples of our simulations with varying magnitudes of perturbation are shown in Figure 2B. With very weak perturbation (RA = 0.001) we observe strong oscillations dominated by a periodic (gamma) component, see Figure 2Ba. As the magnitude of the input perturbation increases, we reach a condition when the high-frequency (gamma) component is constrained to the time segment of increasing perturbation. This shows that the graph theory model can reproduce the respiration-locked modulation of gamma power, i.e., the gamma power increases at the inhalation stage for a suitably selected input signal.

This suggests that the physiological properties of cortical network itself may be sufficient to explain the modulation of gamma power in phase with respiration-locked sensory activity. This is not to say that other factors, such as cortico-thalamic interactions or the action of neuromodulators have no role, but future research will have to determine the nature of their involvement.

Each of these forms of cortical activity appears to have different functions. Oscillatory rhythms that are phase-locked to respiration may help to synchronize large portions of the cortical network and create a temporal alignment for slower processes. The calming effect of controlled, slow and deep breathing could be due to this respiration-locked synchronization of activity across large areas of cortex, an EEG activity pattern commonly observed during meditative states (Dillbeck and Bronson, 1981; Gaylord et al., 1989). Additional evidence of respiration-locked synchronization of cortical oscillatory activity comes from a study of EEG activity during meditation with forced alternate nostril breathing, which caused an increase in interhemispheric beta coherence (Stancák and Kuna, 1994).

Few studies have evaluated cognitive processing as a function of respiratory phase. However, interactions between respiration and non-respiratory functions have been documented in humans and rodents. In humans, for example, phase-locking with respiration has been observed for visual signal detection (Flexman et al., 1974) eye movements (Rittweger and Pöpel, 1998; Rassler and Raabe, 2003), the temporal grouping of pianistic finger movements (Ebert et al., 2002), reaction time to visual (Li et al., 2012) and auditory (Gallego et al., 1991) stimuli, and grip-force (Li and Laskin, 2006). Rassler et al. (1996) reported that response latency, tracking-precision and movement duration of finger movements made to track a visual target showed significant respiratory-phase-dependent differences and that the respiratory-phase-dependence differed between finger flexion and extension movements (Rassler, 2000). In mice, movements of the mystacial whiskers are phase-locked to respiration (Cao et al., 2012; Moore et al., 2013).

Respiration has also been implicated in the modulation of pain perception. Pain-studies in humans showed that pain perception is reduced during inspiration (Arsenault et al., 2013) and that focused slow breathing reduces the perceived severity of pain (Zautra et al., 2010). Other clinical studies have shown that the strength of cortico-spinal communication assessed with transcranial magnetic stimulation (TMS) is modulated in phase with respiration (Li and Rymer, 2011). We suggest that these interactions between respiration and sensory motor processes are mostly caused by respiration-locked fluctuations of ongoing neuronal activity in motor and sensory cortical areas.

In summary, we propose that ongoing neuronal activity of the neocortex is rhythmically modulated by respiration-locked sensory inputs. We predict three emergent patterns of cortical activity that are phase-locked to respiration and are synchronized across large areas of neocortex: (1) neuronal oscillations following the respiratory rhythm; (2) increases in gamma power phase locked to breathing; and (3) the timing of phase transitions in large scale network activity phase locked to respiration. Gamma oscillation power and phase transition timing are strongly implicated in cognitive function, directly linking breathing to cognitive processes. Our findings and hypotheses provide a new perspective of the function of respiration beyond the life-supporting exchange of gases towards a link between the states of the body and mind. This new physiological role of respiration calls for experimental designs to incorporate respiratory information and for future investigations of the interactions between respiration and cognitive, sensory and motor processes.

## Author Contributions

DHH developed the original research concept, participated in data collection and analysis and wrote the manuscript. SSM participated in data collection and analysis and contributed to writing the manuscript. YL and AB-F contributed to the design of the research, to data analysis and to the writing of the manuscript. RR contributed to data collection, analysis and writing of the manuscript. WJF contributed to the design of the research, especially the modeling aspect, and to the writing of the manuscript (WJF passed away before the completion of this manuscript and is included as a posthumous author). JWW participated in the development of experimental designs, coordinated ECoG data collection and contributed to the writing of the manuscript. ACP contributed to the design of the research, to data analysis and to the writing of the manuscript. MR, YS and RK performed the modeling portion of the study. RK and MR wrote the modeling portion of the manuscript and contributed to the writing of the overall manuscript.

## Conflict of Interest Statement

The authors declare that the research was conducted in the absence of any commercial or financial relationships that could be construed as a potential conflict of interest.
